# Agglomerative hierarchical cluster analysis and temporal trend of leprosy indicators in Brazilian states, 2012-2022

**DOI:** 10.1590/0074-02760240163

**Published:** 2025-04-11

**Authors:** Lúcia Rolim Santana de Freitas, Fernanda Fernandez Nóbrega

**Affiliations:** 1 Universidade de Brasília, Faculdade de Medicina, Brasília, DF, Brasil

**Keywords:** leprosy, hierarchical clustering, public health surveillance, time series studies, neglected diseases

## Abstract

**BACKGROUND:**

Leprosy, a neglected tropical disease caused by *Mycobacterium leprae*, presents significant public health challenges in Brazil due to its slow progression, dermato-neurological manifestations, and potential for disability. Understanding leprosy’s spatial distribution and temporal trends is important for effective control and elimination strategies.

**OBJECTIVES:**

This study aimed to identify clusters of leprosy in Brazilian states using agglomerative hierarchical clustering and to analyse their temporal trends from 2012 to 2022.

**METHODS:**

An ecological study was conducted using data from the National System of Notifiable Diseases (SINAN). The agglomerative hierarchical clustering method was used to group states using the new case detection rate (NCDR) of leprosy per 100,000 inhabitants, the proportion of new cases of leprosy with grade 2 physical disability at the time of diagnosis (G2R), and the Gini index, a measure of socioeconomic inequality. Temporal trends within the clusters were assessed using Prais-Winsten regression analysis.

**FINDINGS:**

In the period 2012-2022, 293,030 new cases of leprosy were reported in Brazil. Five distinct clusters were identified. Cluster 4, comprising Mato Grosso and Tocantins, had the highest NCDR and stable temporal trends (APC: 3.2%, 95% CI: -0.1%, 6.7%). Clusters 1 and 3 had the highest proportions of grade 2 disability, indicating late diagnosis. Clusters 4 and 5 had the lowest percentages of individuals with incomplete/complete higher education (7.6% and 7.4%, respectively). Cluster 4 had the highest percentage of individuals with the Diforma clinical form (69.8%) and with cases classified as multibacillary (84.5%).

**MAIN CONCLUSIONS:**

The use of agglomerative hierarchical clustering, a novel application of a non-supervised algorithm in this context, highlighting the integration of multiple epidemiological and socioeconomic variables for a better understanding the dynamics of leprosy transmission in Brazil. Significant variations in the spatial distribution and temporal trends of leprosy were observed across Brazilian states. To improve leprosy surveillance and control in Brazil, targeted interventions are needed, particularly in high-endemicity regions with late diagnosis.

Leprosy is an infectious and contagious disease caused by *Mycobacterium leprae*, which belongs to the group of neglected tropical diseases (NTD).^1^ ^,^ ^2^ ^,^ ^3^ Due to its slow-progressing clinical course and dermato-neurological manifestations, leprosy represents a significant challenge for global public health, especially in countries like Brazil.^4^ The impact of the disease is exacerbated by its disabling potential and the difficulty of early diagnosis, mainly affecting populations in situations of socioeconomic vulnerability.^3^

Leprosy is included among the Sustainable Development Goals (SDGs) as part of the global health policy agenda.^5^ To address the global leprosy burden, the World Health Organization (WHO) launched the “Global Leprosy Strategy 2021-2030”.^4^ This strategy is designed to reduce leprosy prevalence to eliminate leprosy-related disabilities. In Brazil, the “National Strategy for Tackling Hansen’s Disease 2024-2030”^5^ was implemented to align with these WHO goals, aiming to enhance case detection, early treatment, and support for those affected. Together, these global and national initiatives underscore the enduring commitment to combating leprosy and addressing its associated stigma.

The burden of NTDs, including leprosy, is substantial, with 47.9 million disability-adjusted life years (DALYs) attributed to these diseases, predominantly affecting disadvantaged populations.^6^ Despite international efforts to eliminate the disease as a public health problem, included in the global strategy used by the WHO^4^ ^)^ significant challenges remain, especially in endemic countries such as Brazil, India and Indonesia. In 2023, 107,851 (62%) of which occurred in India, 22,773 (13%) in Brazil and 14,376 (8%) in Indonesia. These countries accounted for 83% of total new cases notified globally.^7^

In 2023, Brazil registered a rise of 16.0 per cent in the total of leprosy cases compared to the previous year.^7^ ^,^ ^8^ A comprehensive 20-year ecological and population study in the country revealed worrying trends in leprosy indicators. The analysis showed an increasing temporal trend in leprosy cases between 2001 and 2019, particularly in the North, Northeast and Centre-West regions.^9^

In addition, investigations in São Paulo identified the active circulation of *M. leprae*, indicating ongoing transmission, despite the state being considered non-endemic since 2006.^10^

Analysing the spatial patterns and temporal trends of leprosy in Brazilian states can provide crucial information on the factors that influence the persistence of the disease. Studies in Pernambuco have highlighted high new case detection rate (NCDR), active transmission and clusters of high-risk areas.^11^ Research in São Paulo revealed ongoing transmission scenarios, emphasising the need for surveillance and control measures.^10^ Similarly, research in Minas Gerais identified spatial clusters of leprosy cases, emphasising the importance of targeted interventions in historically endemic regions.^12^ In addition, a study in Rio Grande do Sul showed a profile of low endemicity, indicating variations in the burden of the disease in different states of Brazil.^13^

The identification of clusters through unsupervised techniques and detailed analysis of the temporal trend of the main leprosy indicators in Brazil can reveal critical factors that influence the persistence of the disease in Brazilian states. These findings highlight persistent difficulties in meeting the SDGs, and underscore the ongoing public health challenge posed by leprosy in Brazil, emphasising the need for targeted surveillance and control interventions to address the spatial and spatio-temporal clustering of the disease. The aim of this study is therefore to identify leprosy clusters using the hierarchical agglomerative cluster technique in the Brazilian states, and to describe their temporal trend from 2012 to 2022.

## MATERIALS AND METHODS

This is an ecological study using data from the Information System for Notifiable Diseases (SINAN).^14^ Population data was obtained from the Department of Informatics of the Unified Health System (DATASUS)^15^ and produced by the Brazilian Institute of Geography and Statistics (IBGE).

The study covered all of Brazil’s states, providing a comprehensive view of the geographical and temporal distribution of leprosy at national and state level. Brazil is South America’s largest country (8,510,417.77 km^2^). Divided into five regions (North, Northeast, Midwest, Southeast and South), 26 states (plus the Federal District) and over 5,570 municipalities. In 2022 it had 203.0 million inhabitants.^16^

New cases of leprosy diagnosed in the period 2012-2022 were included in the study as a mode of entry and excluding diagnostic errors. New cases were selected according to place of residence, and diagnosed in the respective years under analysis. To calculate the new case detection rate of leprosy per 100,000 inhabitants, we used the resident population as the denominator based on the 2022 Census and intercensal projections (2012-2021) produced by the IBGE.^16^ The detection rate of new leprosy cases with grade 2 disability was calculated by dividing the number of new cases with grade 2 physical disability at diagnosis (for residents in a specific location within the evaluation year) by the total number of new cases with assessed physical disability in the same location and year.

The variables analysed were: age groups (in years: 0-14, 15-29, 30-49, 50-69, 70-79, 80 and over), sex (male, female), race or skin colour (white, yellow, brown, black and indigenous), education (illiterate, 1st to 4th grade (incomplete or complete), 5th to 8th grade incomplete, primary school complete, high school (incomplete or complete), higher education (incomplete or complete)), number of nerves affected (0, 1, 2 to 5 and > 5), clinical form (indeterminate, tuberculoid, virchowian, diphtheria and unclassified), operational classification (paucibacillary and multibacillary), method of case detection (referral, spontaneous demand, collective examination, examination of contacts and other methods), and year of diagnosis.

The sociodemographic and clinical characteristics of the leprosy cases were analysed using descriptive statistics for absolute and relative frequencies of the selected variables by identified clusters. Continuous variables were summarised as medians and quartiles. Information on missing data is provided for the variables of interest.

To analyse the clusters, we used the agglomerative hierarchical cluster algorithm,^17^ using as indicators the new case detection rate of leprosy per 100,000 inhabitants (TDH), the proportion of new cases of leprosy with grade 2 physical disability at the time of diagnosis (GR2), and the Gini index^18^ as a socioeconomic variable, provided by the Atlas of Human Development in Brazil.^19^ The Gini index was used as a supplementary variable to identify potential socioeconomic disparities between clusters.

Before applying the clustering algorithm, all variables were preprocessed and standardised to normal distributions to ensure comparability across different units and scales. This step was important to prevent any single variable from dominating the clustering results due to differing magnitudes or scales. Hierarchical agglomerative clustering merges data nodes based on their proximity, with the Ward method using the weighted Euclidean distance to minimise variance within clusters.^20^ The Elbow method, commonly used to determine the optimal number of clusters, identifies the point at which adding more clusters does not significantly improve the quality of the clustering.^21^ This approach is important for avoiding overfitting and finding a balance between granularity and cluster quality.

Following the initial cluster analysis of all 27 Brazilian states, the temporal trend analysis was conducted at the cluster level, enabling a clearer depiction of trends of leprosy indicators within each identified group. To highlight temporal changes in leprosy indicators within each cluster, box plots were constructed comparing the distribution of indicators in two distinct time periods (2012-2016) and (2017-2022).

The temporal trend of the indicators was analysed using Prais-Winsten regression,^22^ which takes serial autocorrelation into account. This method estimates parameters controlled by first-order autocorrelation, making it possible to quantify the annual percentage change (APC) of the measures and estimate 95% confidence intervals (95% CI). Trends were classified as increasing (coefficient β_1_ positive and p-value < 0.05), decreasing (coefficient β_1_ negative and p-value < 0.05) or stationary (p-value ≥ 0.05). Statistical significance was defined as p-value < 0.05. The Durbin-Watson (DW) statistic was used to indicate autocorrelated results. Values closer to 2 indicate little to no autocorrelation, while values significantly below 2 suggest positive autocorrelation, and values significantly above 2 suggest negative autocorrelation. The analyses were carried out using R software version 4.3.0 and Anaconda Jupyter Notebook with Python version 3.11.

*Ethics* - This study was conducted exclusively with publicly available secondary data, without identifying the subjects, thus dispensing with the need to submit it to the Research Ethics Committee. The procedures adopted are in accordance with Resolution 466 of 2012 and Resolution 674 of 2022 from the National Health Council, which establishes ethical guidelines for research involving human beings in Brazil.

## RESULTS

**Fig. 1:**
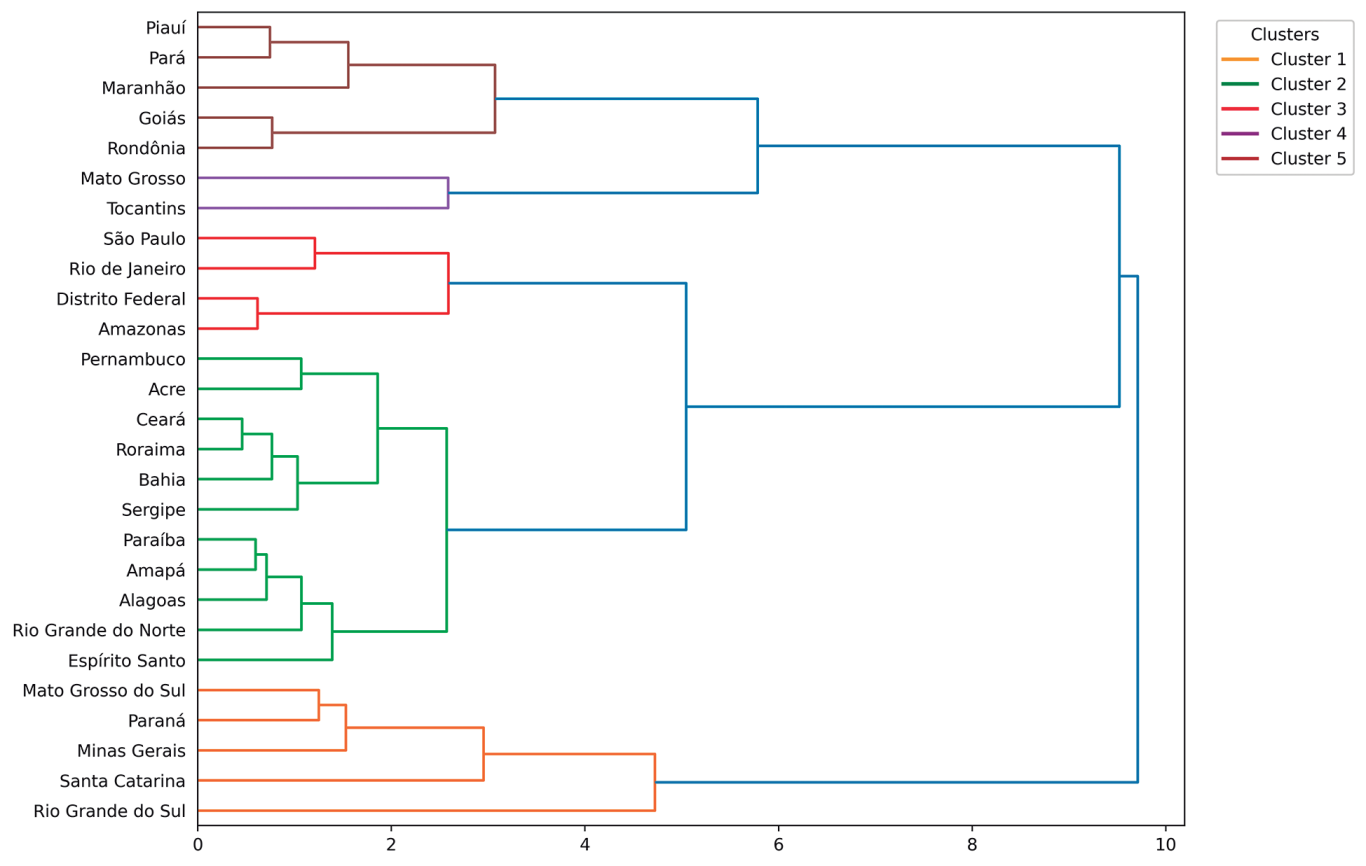
dendrogram of the agglomerative hierarchical cluster analysis for leprosy according to Brazilian states, 2012 to 2022. *The connection distance is shown on the horizontal axis.

In the period 2012-2022, 293,030 new cases of leprosy were reported in Brazil. The agglomerative hierarchical cluster identified five clusters for the 27 Brazilian states. The states that made up each of the clusters are described in the dendrogram (Fig. 1). Cluster 1 included five states (Mato Grosso do Sul, Paraná, Minas Gerais, Santa Catarina and Rio Grande do Sul). Cluster 2 included eleven states located predominantly in the North and Northeast regions; and Cluster 3 included four states (Amazonas, Distrito Federal, Rio de Janeiro and São Paulo). Cluster 4 included the states of Mato Grosso and Tocantins, which have poor leprosy indicators. Cluster 5 included the states of Piauí, Pará, Maranhão, Goiás and Rondônia.

**Fig. 2:**
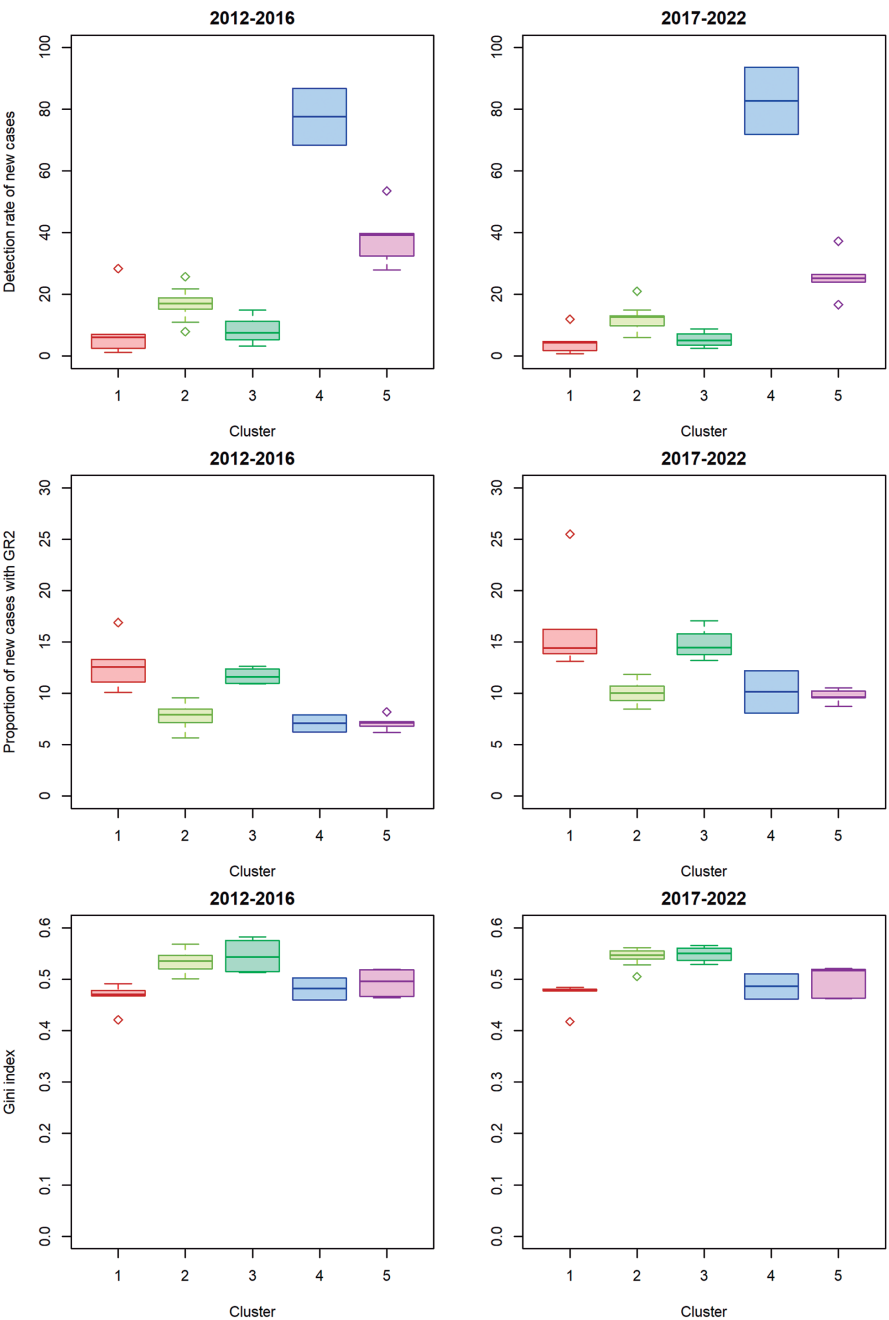
distribution of leprosy epidemiological indicators and Gini index (medians and quartiles) according to the clusters identified by the hierarchical agglomerative method, Brazil, 2012-2016 and 2017-2022.

Cluster 4 stood out markedly from the other clusters in terms of the new case detection rate of leprosy per 100,000 inhabitants (TDH) in the 2012-2016 (median TDH = 77.50 per 100,000 inhab.) and 2017-2022 (median TDH = 82.73 per 100,000 inhab.) periods. In turn, for the proportion of new cases of leprosy with grade 2 physical disability at the time of diagnosis (GR2), Cluster 1 (GR2 = 12.54 in the 2012-2016 period and GR2 = 14.39 in 2017-2022) and Cluster 3 (GR2 = 11.59 in the 2012-2016 period and GR2 = 14.44 in 2017-2022) showed higher median values when compared to the other clusters identified (Fig. 2). For the Gini index, Cluster 2 and Cluster 3 presented the highest median values compared to the other identified clusters (Gini index = 0.54 for 2012-2016 and 0.55 for 2017-2022).

The percentage of individuals aged 0 to 14 was 3.3% for Cluster 1 and 8.1% for Cluster 5 (Table I). Cluster 1 had a higher proportion of individuals with white race/skin colour (47.7%), while Cluster 5 had a higher percentage with brown skin colour (66.8%). In terms of schooling, Clusters 4 and 5 had the lowest percentages of individuals with incomplete/complete higher education (7.6% and 7.4%, respectively). Cluster 4 had the highest percentage of individuals with the Diforma clinical form (69.8%) and with cases classified as multibacillary (84.5%). Clusters 1, 2 and 3 had a higher percentage of cases detected through referral (51.1%; 53.3% and 57.1%, respectively). Clusters 4 and 5 had more cases detected through spontaneous demand (46.0% and 47.5%). The groups were similar in terms of gender among the five clusters identified.

**TABLE I:** Sociodemographic and clinical characteristics of leprosy cases according to clusters identified in Brazil between 2012 and 2022

Variable	Cluster 1		Cluster 2		Cluster 3		Cluster 4		Cluster 5		Total		
	n	%	n	%	n	%	n	%	n	%	n	%
Sex												
Female	11,755	42.7	41,654	46.1	14,002	43.8	22,054	48.0	39,698	41.3	129,163	44.3
Male	15,757	57.3	48,628	53.9	17,982	56.2	23,865	52.0	56,396	58.7	162,628	55.7
Age group (in years)												
0 to 14	911	3.3	5,763	6.4	1,452	4.5	2,583	5.6	7,738	8.1	18,447	6.3
15 to 29	2,992	10.9	13,421	14.9	4,819	15.1	6,719	14.6	17,616	18.3	45,567	15.6
30 to 49	8,888	32.3	31,132	34.5	10,782	33.7	17,859	38.9	33,889	35.3	102,550	35.1
50 to 69	11,380	41.4	30,119	33.4	11,665	36.5	15,595	34.0	28,204	29.4	96,963	33.2
70 to 79	2,565	9.3	7,219	8.0	2,519	7.9	2,467	5.4	6,409	6.7	21,179	7.3
> = 80	777	2.8	2,638	2.9	748	2.3	697	1.5	2,241	2.3	7,101	2.4
Race or skin colour												
White	12,961	47.7	16,793	19.0	13,503	43.0	12,598	27.6	16,557	17.4	72,412	25.1
Yellow	213	0.8	776	0.9	199	0.6	757	1.7	866	0.9	2,811	1.0
Brown	10,414	38.3	55,229	62.5	12,787	40.7	26,297	57.5	63,705	66.8	168,432	58.5
Black	2,888	10.6	11,077	12.5	3,615	11.5	5,346	11.7	12,877	13.5	35,803	12.4
Indigenous	124	0.5	319	0.4	290	0.9	280	0.6	293	0.3	1,306	0.5
Ignored	590	2.2	4,225	4.8	1,047	3.3	446	1.0	1,009	1.1	7,317	2.5
Education												
Illiterate	10,815	42.9	31,853	39.6	9,290	31.4	15,343	35.2	39,764	44.3	107,065	39.9
1st to 4th grade (incomplete or complete)	3,727	14.8	11,675	14.5	4,625	15.7	7,361	16.9	15,526	17.3	42,914	16.0
5th to 8th grade incomplete	1,568	6.2	4,263	5.3	2,479	8.4	3,144	7.2	5,555	6.2	17,009	6.3
Primary school complete	3,999	15.8	14,927	18.5	6,759	22.9	10,916	25.1	18,503	20.6	55,104	20.5
High school (incomplete or complete)	1,200	4.8	3,634	4.5	1,813	6.1	3,486	8.0	3,724	4.2	13,857	5.2
Higher education (incomplete or complete)	3,932	15.6	14,167	17.6	4,595	15.5	3,308	7.6	6,651	7.4	32,653	12.2
Number of nerves affected												
0	12,147	44.2	46,855	51.9	14,333	44.8	11,609	25.3	48,845	50.8	133,789	45.9
1	3,328	12.1	8,983	10.0	3,556	11.1	5,092	11.1	10,860	11.3	31,819	10.9
2 a 5	8,096	29.4	14,384	15.9	8,588	26.9	20,604	44.9	22,751	23.7	74,423	25.5
> 5	3,942	14.3	20,070	22.2	5,508	17.2	8,615	18.8	13,641	14.2	51,776	17.7
Clinical form												
Undetermined	2,919	10.9	14,233	16.6	3,983	12.8	4,558	10.1	13,807	14.7	39,500	14.0
Tuberculoid	3,657	13.6	17,823	20.8	6,335	20.4	3,576	7.9	11,919	12.7	43,310	15.3
Virchowian	7,074	26.3	15,221	17.7	7,824	25.2	3,794	8.4	14,869	15.8	48,782	17.3
Diforma	12,055	44.8	30,880	36.0	11,671	37.6	31,425	69.8	49,046	52.3	135,077	47.8
Not classified	1,208	4.5	7,633	8.9	1,242	4.0	1,688	3.8	4,213	4.5	15,984	5.7
Operational classification												
Paucibacillary	6,175	22.5	32,342	35.8	9,847	30.8	7,108	15.5	25,166	26.2	80,638	27.6
Multibacillary	21,332	77.6	57,930	64.2	22,132	69.2	38,812	84.5	70,929	73.8	211,135	72.4
Case detection mode												
Spontaneous demand	13,928	51.1	47,568	53.3	18,133	57.1	13,075	28.7	39,602	41.5	132,306	45.8
Referral	9,074	33.3	31,684	35.5	9,443	29.7	20,915	46.0	45,300	47.5	116,416	40.3
Collective examination	507	1.9	2,680	3.0	1,127	3.6	2,684	5.9	3,940	4.1	10,938	3.8
Contact examination	3,111	11.4	5,066	5.7	2,572	8.1	7,902	17.4	5,016	5.3	23,667	8.2
Other modes	576	2.1	1,833	2.1	409	1.3	860	1.9	1,379	1.5	5,057	1.8
Ignored	70	0.3	345	0.4	84	0.3	84	0.2	144	0.2	727	0.3

In the period 2012-2022, there was a decreasing trend for the new case detection rate of leprosy per 100,000 inhabitants for Cluster 1 (APC: -7.81%, 95%CI: -6.12% to -9.47%), Cluster 2 (APC: -6.07%, 95%CI: -7.92% to -4.19%), Cluster 3 (APC: -7.11%, 95%CI: -8.30% to -5.91%) and Cluster 5 (APC: -8.32%, 95%CI: - 10.58% to -5.99%) (Table II). Unlike the other clusters, the NCDR for Cluster 4 was stable in the study period (APC: 3.2%, 95%CI: -0.1% to 6.7%). Analysing the Durbin-Watson statistic revealed variations in the autocorrelation of the residuals between the different clusters (Table II). The models fitted for Cluster 2 (1.85), Cluster 4 (2.08) and Cluster 5 (1.92) showed little or no autocorrelation, indicating that the models fitted for these clusters are appropriate. On the other hand, the models fitted for Cluster 1 (1.23) and Cluster 3 (1.67) suggest positive autocorrelation. During the period (2012-2022), there was a increasing trend for the proportion of new cases of leprosy with grade 2 physical disability for all identified clusters (Table III). Analysing the Durbin-Watson statistic, the models fitted for Cluster 1 (1.68), Cluster 2 (1.59) and Cluster 5 (1.49) suggest positive autocorrelation.

**TABLE II:** Annual percentage change (APC) and trend in the new case detection rate of leprosy per 100,000 inhabitants, according to identified clusters, Brazil, 2012 to 2022

Cluster	APC	CI (95%)	p-value	Durbin-Watson statistic	Trend
1	-7.81	(-6.12 to -9.47)	< 0.001	1.99	Decreasing
2	-6.07	(-7.92 to -4.19)	< 0.001	2.08	Decreasing
3	-7.11	(-8.30 to -5.91)	< 0.001	2.00	Decreasing
4	-2.24	(-9.60 to 5.73)	0.530	1.23	Stationary
5	-8.32	(-10.58 to -5.99)	< 0.001	1.89	Decreasing
Brazil	-6.40	(-8.86 to -3.87)	< 0.001	1.85	Decreasing

CI (95%): 95% confidence interval.

**TABLE III:** Annual percentage change (APC) and trend in the proportion of new leprosy cases with grade 2 physical disability, according to identified clusters, Brazil, 2012 to 2022

Cluster	APC	CI (95%)	p-value	Durbin-Watson statistic	Trend
1	5,42	(4,81 to 6,02)	< 0,001	1,68	Increasing
2	4,83	(3,41 to 6,28)	< 0,001	1,59	Increasing
3	4,90	(3,41 to 6,40)	< 0,001	1,75	Increasing
4	6,15	(2,87 to 9,56)	0,002	2,07	Increasing
5	4,55	(2,79 to 6,33)	< 0,001	1,49	Increasing
Brazil	4,71	(3,30 to 6,14)	< 0,001	1,93	Increasing

CI (95%): 95% confidence interval.

## DISCUSSION

This study used current epidemiological indicators and temporal analysis to characterise the distribution of cases and the trend of the endemic in the identified clusters. The agglomerative hierarchical cluster analysis revealed five clusters of Brazilian states with distinct epidemiological characteristics, offering a detailed view of the distribution of leprosy in Brazil in the period 2012-2022.

Our study found that the Cluster 4 (which corresponds to the states of Mato Grosso and Tocantins) stood out with the highest NCDR. These states had high NCDR, possibly due to the low coverage of health services and the high endemicity of leprosy. Clusters 1 and 3 had the highest proportions of cases with grade 2 physical disability at diagnosis. This finding suggests late diagnosis in these states, indicating the need for improvements in surveillance and early detection of the disease.

The high GR2 values in Clusters 1 (Mato Grosso do Sul, Paraná, Minas Gerais, Santa Catarina and Rio Grande do Sul) and Cluster 3 (Amazonas, Distrito Federal, Rio de Janeiro and São Paulo) indicate a significant problem of late diagnosis. This suggests the need to strengthen surveillance systems and health education strategies to promote early detection of the disease. The literature suggests that high GR2 values are associated with late diagnosis, where individuals are not diagnosed until they show advanced signs of the disease, resulting in higher rates of disability.^23^

The findings of this study are consistent with the existing literature on leprosy in Brazil, which identifies areas of high prevalence and problems with late diagnosis. Previous studies have also highlighted the persistence of leprosy in regions such as the North and Northeast, associated with socioeconomic factors and limited access to health services.^24^ ^,^ ^25^ Cluster 5 (which includes the states of Piauí, Pará, Maranhão, Goiás and Rondônia) was found to have a higher proportion of leprosy in children and a higher proportion of individuals with brown race or skin colour. These findings are consistent with previous studies highlighting the vulnerability of children in areas of high endemicity and reflect the demographic composition of the North and Northeast regions of Brazil, where leprosy is more prevalent.^11^ ^,^ ^23^ The higher ratio observed between low levels of schooling and higher incidence of leprosy in Clusters 4 and 5 is supported by literature associating leprosy with social determinants of health, including low schooling, poverty and limited access to health services.^26^ Cluster 4, characterised by the predominance of the clinical form and multibacillary cases of leprosy, is in line with findings indicating that these forms are prevalent in regions with continuous transmission and late diagnosis.^27^ The clinical form differs and multibacillary cases are linked to a higher bacillary load, increasing the potential for transmission of the disease.^28^

There was a significant decreasing trend in the NCDR of leprosy for clusters 1, 2, 3 and 5. Unlike the other clusters, the NCDR in Cluster 4 remained stable throughout the study period. The results of this study are consistent with existing literature, which also points to a decreasing trend in the incidence of leprosy in various regions of Brazil, attributed to improvements in public health programmes and efforts to control the disease.^29^ However, the stability in Cluster 4 is a worrying finding that requires further investigation and intervention. An increase in the trend was observed for the proportion of new cases of leprosy with grade 2 physical disability for all identified clusters.

This study has some limitations that are worth mentioning. These include the inherent limitations of studies based on secondary data, which may present underreporting and variability in the quality of epidemiological data between states. Discrepancies in diagnostic methods and training for early detection between regions can influence the results, leading to a potential underestimation or overestimation of detection rates and proportions of physical disability. To minimise these limitations, the statistical analysis used hierarchical clusters to group together states with similar epidemiological characteristics, considering aggregated data (2012-2016) and (2017-2022), seeking to improve the accuracy of the results. It is important to note that the underreporting is more likely in the early stages of the disease. This factor could partially explain the observed increase in the proportion of new cases presenting with grade 2 disabilities (a condition in which underreporting is likely less common) alongside a decrease in overall leprosy detection rates (where underreporting is expected to be high). Variations in underreporting indicators over time may introduce a significant detection bias in trend analysis, and this assumption warrants further investigation.

This study offers several important contributions to the understanding of leprosy in Brazil: 1) The identification of five distinct clusters of Brazilian states with specific epidemiological characteristics provides critical information for the formulation of public health policies and targeted interventions; 2) The study highlights the need for differentiated interventions in regions such as Mato Grosso and Tocantins, where high NCDR were identified; 3) The findings can guide the formulation of more effective and targeted health policies focused on improving early detection and treatment of leprosy, especially in endemic states; 4) The combination of current epidemiological indicators with temporal analysis provides a comprehensive view of the evolution of leprosy in Brazil over a decade, allowing for a better understanding of the trends and dynamics of the disease.

To address the issues raised by this study and make progress in eliminating leprosy as a public health problem, it is important to strengthen epidemiological surveillance to ensure early detection of new cases and continuous monitoring of disease trends. This includes improving health information systems and training health professionals to make accurate and timely diagnoses. Other strategies include expanding the coverage of health services in endemic regions, and guaranteeing access to diagnosis and adequate treatment.

Future studies in leprosy research should focus on exploring zoonotic transmission, evaluating the effectiveness of specific interventions and understanding the dynamics of the disease in various socioeconomic contexts. Research has shown that leprosy is significantly influenced by social determinants such as age, poor sanitary conditions, low levels of education and food insecurity.^30^ In addition, successful leprosy control programmes have implemented strategies such as BCG vaccination, active case finding and adherence to multi-drug therapy, leading to a decline in incidence in low-endemic countries.^31^ Understanding the socioeconomic factors that affect leprosy, the potential zoonotic sources of transmission and the effectiveness of interventions will be crucial in developing policies and targeted interventions to fulfil the SDGs and reduce the disease burden globally.

This study highlights the complexity of the spatial distribution of leprosy in Brazil and the need for differentiated approaches to its control. Identifying clusters of states with distinct epidemiological characteristics provides a solid basis for developing more effective and targeted public health policies. The combination of improved surveillance, health education, expanded coverage of health services, and ongoing research is essential to reducing the leprosy burden in Brazil and making progress towards eliminating the disease. The persistence of leprosy in certain regions of Brazil reflects socioeconomic inequalities and limited access to health services. Addressing these challenges requires a joint effort by governments, health organisations, communities and researchers. Only with an integrated and multifaceted approach will it be possible to control leprosy and improve the quality of life of those affected by the disease.

This study brings significant new contributions to the field of public health, particularly in the use of agglomerative hierarchical clustering, an unsupervised approach that allows the identification of complex epidemiological patterns. By identifying clusters of states with specific epidemiological characteristics, this study provides clear evidence for the need for targeted interventions, especially in regions such as Mato Grosso and Tocantins (Cluster 4), where high NCDR indicates a continued challenge for leprosy control.

These results provide information on the dynamics of leprosy in Brazil, highlighting the different trends in the NCDR between the clusters. The reduction in leprosy in some regions is a positive result, but it requires ongoing surveillance, diagnosis and treatment actions to prevent a recrudescence of the disease. More in-depth regional investigations and a multi-sectoral approach that includes actions in the areas of health, education, social assistance and community participation are essential for effective leprosy control in Brazil. The identification of distinct clusters, characterised by varying levels of disease burden and socioeconomic factors, highlights the importance of targeted interventions in high-endemic regions. The analysis of temporal trends reveals the need for sustained efforts to improve early case detection. These findings are particularly relevant for the formulation of public health strategies aimed at improving early detection and treatment of leprosy. Strategies such as expanding health service coverage in endemic regions, strengthening epidemiological surveillance systems, and health education, and targeting more vulnerable populations, are essential to mitigate the impacts of the disease.

In conclusion, the findings of this study reinforce the need for targeted interventions and effective health policies to tackle leprosy in a comprehensive and sustainable manner. With the implementation of the proposed recommendations, significant progress is expected in the fight against leprosy, promoting the health and well-being of affected populations.
